# Planetary Health Diet Adherence and Medication Use in Older Adults with Chronic Kidney Disease: A Cross-Sectional Study

**DOI:** 10.3390/geriatrics11010017

**Published:** 2026-02-05

**Authors:** Luca Soraci, Guido Gembillo, Maria Elsa Gambuzza, Edlin Villalta Savedra, Chiara Chinigò, Elvira Filicetti, Mara Volpentesta, Giada Ida Greco, Domenico Santoro, Andrea Corsonello

**Affiliations:** 1 Unit of Geriatric Medicine, Italian National Research Center on Aging (IRCCS INRCA), 87100 Cosenza, Italy; l.soraci@inrca.it (L.S.); e.filicetti@inrca.it (E.F.); m.volpentesta@inrca.it (M.V.); g.greco@inrca.it (G.I.G.); a.corsonello@inrca.it (A.C.); 2 Unit of Nephrology and Dialysis, Department of Clinical and Experimental Medicine, University of Messina, 98125 Messina, Italy; guidogembillo@live.it (G.G.); dsantoro@unime.it (D.S.); 3 Clinical Pathology Laboratory, Ospedale Ignazio Barone Romeo, 98066 Patti, Italy; 4 Independent Researcher, 87100 Cosenza, Italy; edlinvillalta@gmail.com; 5 Centre for Biostatistics and Applied Geriatric Clinical Epidemiology, Italian National Research Center on Aging (IRCCS INRCA), 87100 Cosenza, Italy; chiarachini95@gmail.com; 6 Department of Pharmacy, Health and Nutritional Sciences, School of Medicine and Digital Technologies, University of Calabria, 87036 Rende, Italy

**Keywords:** chronic kidney disease, planetary health diet, polypharmacy, medication burden, older adults, dietary patterns, nephrotoxic medications, plant-based diet

## Abstract

**Background/Objectives**: Chronic kidney disease (CKD) in older adults is frequently accompanied by substantial medication burden, increasing risks of adverse drug events and poor adherence. The Planetary Health Diet Index (PHDI), emphasizing plant-based foods and sustainable dietary patterns, may improve cardiometabolic health and reduce medication requirements. This study examined the association between PHD adherence as measured by the PHDI and medication burden among older adults with CKD. **Methods**: We analyzed cross-sectional data from the National Health and Nutrition Examination Survey (NHANES) 2003–2018 cycles. Older individuals aged ≥ 65 years with CKD (estimated glomerular filtration rate < 60 mL/min/1.73 m^2^ or albumin-to-creatinine ratio > 30 mg/g) at the baseline visit were included (*n* = 3161). PHDI scores (0–150) were calculated from two consecutive 24 h dietary recalls. Medication burden was assessed as the total prescription medication count and frequency of individual classes. Multivariable Poisson regression models evaluated associations between PHDI score and number of prescribed medications, adjusting for sociodemographic, lifestyle, and clinical covariates; logistic regression models were used to evaluate the association between PHDI score and specific medication classes. **Results**: Mean (SD) age was 75.0 (5.5) years; mean PHDI score was 62.4 (18.7). Participants in the highest PHDI tertile had significantly lower medication burden compared to the lowest tertile. In fully adjusted Poisson regression models, each 10-point increase in PHDI score was associated with 3% fewer medications (RR: 0.97, 95% CI: 0.96–0.99, *p* = 0.011). Participants in the highest PHDI tertile had 8% fewer medications compared to the lowest tertile (RR: 0.92, 95% CI: 0.87–0.98, *p* = 0.013). Higher PHDI scores were significantly associated with lower odds of proton pump inhibitor use (OR: 0.86, 95% CI: 0.79–0.94 per 10-point increase) and nonsteroidal anti-inflammatory drug prescription (OR: 0.86, 95% CI: 0.76–0.97 per 10-point increase). Participants in the highest PHDI tertile had 34% lower odds of PPI use (OR: 0.66, 95% CI: 0.49–0.89) and nonsignificant lower odds of NSAID use (OR: 0.67, 95% CI: 0.40–1.11) compared to those in the lowest tertile. **Conclusions**: Higher PHDI adherence was independently associated with lower medication burden in older adults with CKD. These findings suggest that plant-forward, sustainable dietary patterns may reduce pharmacological complexity in this vulnerable population. Prospective studies are needed to assess causality and clinical implementation strategies.

## 1. Introduction

Chronic kidney disease (CKD) affects approximately 15% of adults worldwide [1], with prevalence exceeding 40% among individuals aged 70 years and older [2]. CKD patients experience extremely high rates of comorbid conditions, including hypertension, diabetes, anemia, and cardiovascular diseases, which substantially increase mortality and complicate treatment [3,4,5]. A multidisciplinary approach is needed to manage overlapping pharmacologic risks [3]. Older adults with CKD commonly experience polypharmacy, defined as the concurrent use of five or more medications, with some patients requiring 10 or more daily prescriptions [6,7]. This medication burden is associated with increased risks of adverse drug reactions, drug–drug interactions, falls, cognitive impairment, poor medication adherence, and greater healthcare costs [8,9]. The medication classes commonly prescribed to CKD patients include antihypertensives (particularly renin-angiotensin-aldosterone system inhibitors), diuretics, phosphate binders, erythropoiesis-stimulating agents, iron supplements, vitamin D analogs, sodium bicarbonate for metabolic acidosis, statins, antiplatelet agents, and hypoglycemic medications [3,10]. Each additional medication increases the complexity of disease management and the potential for treatment-related complications, particularly in the geriatric population where altered pharmacokinetics and pharmacodynamics further complicate therapeutic decision-making [11,12].

Dietary interventions represent a potentially modifiable factor that could improve health outcomes and reduce medication requirements in CKD. Traditional renal diets have emphasized restriction of protein, sodium, potassium, and phosphorus; however, these restrictive approaches may contribute to malnutrition and poor dietary quality in older adults [13]. The Planetary Health Diet (PHD), developed by the EAT-Lancet Commission on Food, Planet, and Health, proposes an alternative framework emphasizing whole grains, fruits, vegetables, legumes, nuts, and unsaturated oils, with moderate amounts of fish and poultry and minimal red meat and processed foods [14]. The Planetary Health Diet Index (PHDI) quantifies adherence to PHD principles across 15 food components, with scores ranging from 0 to 150 [15,16]. Higher PHDI scores reflect greater consumption of health-promoting plant foods and lower intake of animal-based and processed products. Recent epidemiological studies have demonstrated associations between higher PHDI adherence and reduced risks of type 2 diabetes, cardiovascular disease, cancer, and all-cause mortality in general populations [17,18,19]. The mechanisms underlying these benefits likely involve improved glycemic control, blood pressure regulation, lipid profiles, anti-inflammatory effects, antioxidant capacity, and favorable modulation of the gut microbiome [20,21].

In the context of CKD, the PHDI may offer specific advantages [22]. In prospective cohort data, higher adherence to plant-based diet was associated with slower eGFR decline and decreased incidence of CKD [23,24]. More recently, PHDI-focused analyses in middle-aged/older adults have reported that greater adherence to a planetary health diet score is associated with lower CKD risk, supporting the kidney relevance of the PHDI construct [25]. Several mechanisms may explain these effects: high dietary fiber intake promotes production of short-chain fatty acids by gut bacteria, which may reduce generation of uremic toxins [26]. The alkaline ash of plant foods may help ameliorate metabolic acidosis, a recognized driver of tubulointerstitial injury and CKD progression; in a randomized trial, treatment with base-producing fruits and vegetables improved acid–base status and was associated with less eGFR decline compared with usual care, with additional cardiometabolic benefits [27]. Additionally, the cardiovascular and metabolic benefits of plant-predominant diets may translate into better blood pressure and glycemic control, both of which are important for slowing the progression of CKD [24].

Despite the theoretical rationale and emerging evidence for plant-based dietary patterns in CKD management, no studies have specifically examined the relationship between PHD adherence and medication burden in older adults with CKD. Given the unique vulnerabilities of this population to both CKD progression and medication-related adverse events, understanding whether dietary quality influences pharmacological complexity could inform clinical practice and public health recommendations.

Therefore, the objective of this study was to examine the association between PHD adherence and medication burden among older adults with CKD using nationally representative data from the National Health and Nutrition Examination Survey (NHANES) 2003–2018. We hypothesized that higher PHDI scores would be associated with lower medication counts, independent of traditional confounding factors. Secondary objectives included examining whether higher PHDI scores were associated with decreased prescription of specific medication classes commonly prescribed in CKD as well as potentially nephrotoxic medications, such as proton pump inhibitors (PPIs) and nonsteroidal anti-inflammatory drugs (NSAIDs).

## 2. Materials and Methods

### 2.1. Study Population and Design

This cross-sectional study utilized data from eight consecutive cycles of the National Health and Nutrition Examination Survey (NHANES): 2003–2018. NHANES is a nationally representative survey of the non-institutionalized civilian U.S. population conducted by the National Center for Health Statistics (NCHS) of the Centers for Disease Control and Prevention (CDC) [28]. The survey employs a complex, stratified, multistage probability sampling design to ensure national representation. All NHANES protocols were approved by the NCHS Research Ethics Review Board, and all participants provided written informed consent.

Starting from 61,872 participants undergoing two consecutive 24 h dietary recall visits, we selected 3181 participants aged 65 years and older and with a diagnosis of CKD at the baseline visit. After removing 20 individuals with extreme caloric intake (having a total daily energy < 500 Kcal or ≥8000 Kcal), we obtained a final analytic sample of 3161 individuals to be included in the study.

### 2.2. PHDI

Dietary intake was assessed using two non-consecutive 24 h dietary recalls, collected by trained interviewers using the United States Department of Agriculture (USDA) Automated Multiple-Pass Method. Day 1 data were collected in person at the Mobile Examination Center; day 2 data were collected via telephone interviews 3–10 days later, according to methodology presented elsewhere [29]. The average of the two days was used to estimate daily intake of foods and nutrients and total energy intake (TEI) in Kcal/day. Dietary data were then linked to the Food Patterns Equivalents Database, which categorizes foods into the 37 USDA Food Pattern Components, using a food composition table [30]. Food patterns equivalents were then converted in grams/day according to standardized conversion units. Adherence to the PHD was then assessed using the PHDI scoring system proposed by the EAT-Lancet Commission [16] and further adapted to the US population [31]. The 15 PHDI components and criteria used to calculate PHDI are shown in Supplementary Table S1. In brief, each of the 15 food components was assigned a score ranging from 0 (lowest adherence) to 10 (highest adherence) based on recommended intake ranges. For example, a value of 10 was awarded when component intake met or exceeded the EAT-Lancet recommended minimum (for adequacy components) or stayed below the upper threshold (for moderation components). Partial points were proportionally assigned between minimum and maximum thresholds. The points of each of the 15 food components were then summed to obtain an overall PHDI score, ranging from 0 to 150, and representing adherence to the diet, with higher scores indicating greater alignment with the planetary health dietary model.

### 2.3. CKD Definition

Kidney function was assessed using two standard biomarkers: the estimated glomerular filtration rate (eGFR) and the urinary albumin-to-creatinine ratio (ACR). eGFR was calculated by using the race-free CKD-EPI 2021 equation [32], while ACR was determined from spot urine samples by measuring urinary albumin and creatinine concentrations. CKD was defined as the presence of eGFR < 60 mL/min/1.73 m^2^ or ACR > 30 mg/g [32]

### 2.4. Study Covariates

Demographic variables included age, sex, race/ethnicity, and poverty income ratio as a measure of socioeconomic status. Poverty income ratio represents the ratio of family income to the federal poverty threshold, with values < 1.0 indicating income below the poverty level [33].

Lifestyle factors included smoking status and alcohol consumption. Smoking status was assessed using standardized NHANES questionnaire items. Participants were classified as current smokers if they reported having smoked at least 100 cigarettes in their lifetime and answered either “every day” or “some days” to the question “Do you now smoke cigarettes?”. Participants who reported smoking at least 100 cigarettes in their lifetime, but who were not currently smoking, were classified as former smokers, while those who reported smoking fewer than 100 cigarettes in their lifetime were classified as never smokers. For the purpose of this analysis, current and former smokers were combined into a single smoking category. Alcohol consumption was defined as consuming ≥12 alcoholic drinks in the past year.

Comorbid conditions were assessed through self-report of physician diagnosis and included hypertension, diabetes mellitus, cardiovascular disease (congestive heart failure or coronary artery disease), stroke, cancer (any malignancy), and chronic obstructive pulmonary disease (COPD). Diabetes mellitus diagnosis was supplemented with use of laboratory glycated hemoglobin (HbA1c) ≥ 6.5%.

Other laboratory measurements included fasting glucose, high-density lipoprotein cholesterol (HDL-C), low-density lipoprotein cholesterol (LDL-C), C-reactive protein (CRP), and uric acid. All laboratory analyses were performed at standardized centers following NHANES protocols.

### 2.5. Medication Assessment

Prescription medication data were collected during the household interview, as previously reported [34]. Participants were asked to show the interviewer all prescription medications taken in the past 30 days. Interviewers recorded the name of each medication from the container. Medication names were matched to their therapeutic class using the Lexicon Plus database, a proprietary database of Cerner Multum, Inc. Total medication count was defined as the number of unique prescription medications currently used. Polypharmacy was defined as the concurrent use of five or more prescription medications. Specific medication classes of interest included angiotensin-converting enzyme inhibitors (ACEi), angiotensin receptor blockers (ARBs), beta-blockers, calcium channel blockers (CCBs), statins, glucose-lowering medications, urate-lowering medications, antiplatelet medications, and phosphate binders. Potentially nephrotoxic medications assessed included PPIs and NSAIDs.

### 2.6. Statistical Analysis

All analyses incorporated NHANES survey weights to account for the complex survey design, including stratification, clustering, and non-response, ensuring nationally representative estimates. Because dietary exposure was derived from two 24 h dietary recalls, all analyses were conducted using the NHANES dietary two-day sample weights, which are specifically designed for participants with complete two-day dietary recall data. As the present analysis combined eight consecutive NHANES cycles (2003–2018), the original two-day sample weights were divided by eight, in accordance with National Center for Health Statistics analytic guidelines, to obtain appropriate nationally representative estimates across the pooled survey period.

Survey-weighted descriptive statistics were calculated for baseline characteristics overall and stratified by PHDI tertiles. PHDI tertiles were defined according to the weighted distribution of the score, with T1 representing the lowest adherence, T2 intermediate adherence, and T3 the highest adherence to the PHD. Continuous variables were presented as means with standard deviations (SD) or medians with interquartile ranges (IQR), as appropriate. Categorical variables were expressed as frequencies and weighted percentages. Comparisons across PHDI tertiles were conducted using survey-weighted one-way ANOVA for normally distributed continuous variables, survey-weighted Kruskal–Wallis test for non-normally distributed continuous variables, and Rao-Scott adjusted chi-square test for categorical variables.

The association between PHDI and medication burden was examined using survey-weighted Poisson regression models with robust variance estimation, given the count nature of medication number with overdispersion. PHDI was analyzed both as a continuous variable (per 10-point increase) and categorically (tertiles). Two sequential models were constructed: Model A adjusted for age, sex, race/ethnicity, and poverty income ratio; Model B additionally adjusted for smoking status, alcohol consumption, hypertension, diabetes mellitus, cardiovascular disease, stroke, COPD, cancer, eGFR, and ACR.

Survey-weighted multivariable logistic regression models were used to assess associations between PHDI and use of specific medication classes, including PPIs and NSAIDs. Models were adjusted for the same covariates as Model B in the Poisson regression analyses, with additional mutual adjustment for PPI use when examining NSAIDs, and vice versa.

All statistical tests were two-sided, with *p* < 0.05 considered statistically significant. Analyses were performed using R software version 4.3.0 (R Foundation for Statistical Computing, Vienna, Austria), incorporating the survey package for complex survey design adjustments.

## 3. Results

### 3.1. Baseline Characteristics of the Study Population

The final analytical sample included 3161 older adults with CKD, representative of the U.S. population. Baseline characteristics of the study population, overall and stratified by PHDI tertiles, are shown in Table 1.

Overall, the mean (SD) age was 75.0 (5.5) years, and 56.4% of participants were women. The majority were white (80.3%), and the mean (SD) poverty income ratio was 2.7 (1.5). The mean (SD) PHDI score was 76.6 (12.9). Across increasing PHDI tertiles, defined as T1 (lowest PHDI adherence), T2 (intermediate PHDI adherence), and T3 (highest PHDI adherence), participants differed significantly in several sociodemographic and lifestyle characteristics. Individuals in the highest PHDI score tertile were slightly older and were more likely to be female compared with those in the lowest tertile (65.6% vs. 46.3%, *p* < 0.001). Higher PHDI adherence was also associated with a more favorable socio-economic condition, represented by higher poverty income ratios (*p* < 0.001) and a lower prevalence of smoking (45.2% in T3 vs. 61.4% in T1, *p* < 0.001). Alcohol consumption did not differ significantly across tertiles (*p* = 0.126).

With respect to comorbidities, the prevalence of hypertension, diabetes, cardiovascular disease, stroke, and cancer was high in this older CKD population but did not differ significantly across PHDI tertiles (all *p* > 0.05). Mean (SD) eGFR was 58.3 (18.7) mL/min/1.73 m^2^ and was comparable across tertiles (*p* = 0.160). Similarly, median ACR did not vary significantly by PHDI adherence (*p* = 0.769).

Several metabolic and biochemical markers differed across tertiles. Higher PHDI adherence was associated with lower mean fasting glucose levels (*p* = 0.017) and lower serum uric acid concentrations (*p* = 0.004). Participants in the highest PHDI tertile also exhibited a more favorable lipid profile, with significantly higher HDL-cholesterol levels compared with those in the lowest tertile (57.1 vs. 51.0 mg/dL, *p* < 0.001). Mean HbA1c showed a borderline decreasing trend across tertiles (*p* = 0.058), while LDL-cholesterol and C-reactive protein concentrations did not differ significantly by PHDI tertile.

Table 2 summarizes medication burden and patterns of medication use in the overall study population and across PHDI tertiles.

Overall, participants used a mean (SD) of 5.2 (3.3) medications, and 53.9% met criteria for polypharmacy. The average number of medications differed significantly across PHDI tertiles, with participants in the highest PHDI tertile using fewer medications compared with those in the lowest tertile (4.9 vs. 5.4 medications; *p* = 0.025). The prevalence of polypharmacy decreased across increasing PHDI tertiles, although this trend was not statistically significant.

With respect to cardiovascular medications, the use of ACEi, ARBs, beta blockers, CCBs, and statins was common but did not vary significantly by PHDI tertile (all *p* > 0.05). Similarly, no significant differences across tertiles were observed for the use of glucose-lowering agents, urate-lowering agents, antiplatelet agents, or phosphate binders, the latter being infrequently used in the overall population.

In contrast, the use of selected medication classes differed across PHDI tertiles. In particular, PPI use was significantly lower among participants in the highest PHDI tertile compared with those in the lower tertiles (16.7% in T3 vs. 21.5% in T1; *p* = 0.007). Similarly, the prevalence of NSAID use decreased across increasing PHDI tertiles, with the lowest use observed in the highest tertile (5.2% in T3 vs. 8.5% in T1; *p* = 0.003).

### 3.2. Association Between PHDI Score and Number of Medications

**Figure 1:**
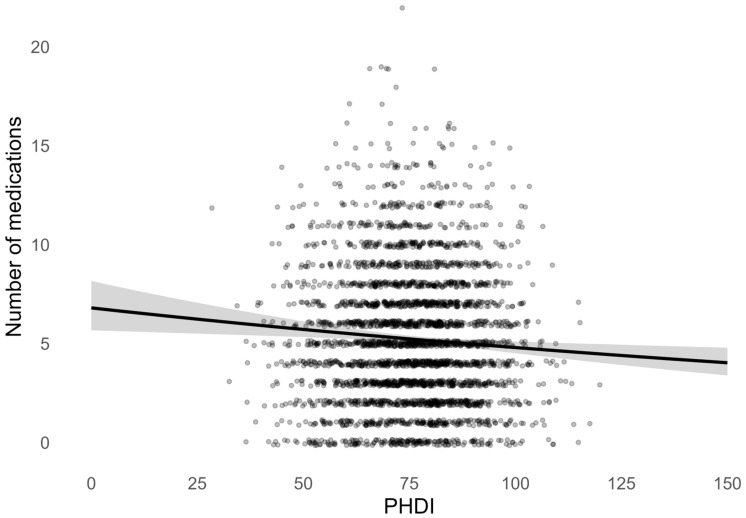
Survey-weighted Poisson regression curve showing the association between PHDI score and number of medications.

As shown in Figure 1, higher PHDI scores were associated with a lower medication burden. The survey-weighted unadjusted Poisson regression curve demonstrates a gradual inverse relationship between PHDI score and the expected number of medications, with the predicted mean number of drugs decreasing steadily across the observed range of PHDI values. This association was evident despite substantial inter-individual variability in medication counts, particularly at lower PHDI scores. Each 10-point increase in PHDI score (PHDI-10) was associated with a relative 4% reduction in the number of medications, supporting a dose–response relationship between diet quality and medication burden (RR, 95% CI: 0.96, 0.94–0.99, *p* = 0.004).

These preliminary findings were confirmed in survey-weighted multivariate Poisson regression models (Table 3).

When PHDI was modeled as a continuous variable (per 10-point increase), PHDI-10 was associated with a 3% lower number of medications in both models. This association was statistically significant in the minimally adjusted model (Model A: RR = 0.97, 95% CI 0.94–0.99; *p* = 0.002) and remained significant after further adjustment for additional covariates in Model B (RR = 0.97, 95% CI 0.96–0.99; *p* = 0.011), indicating robustness to confounding.

When PHDI was analyzed in tertiles, participants in the highest tertile (T3) had a significantly lower number of medications compared with those in the lowest tertile (T1). In Model A, membership in T3 was associated with a 10% lower medication count (RR = 0.90, 95% CI 0.83–0.96; *p* = 0.003), and this association persisted after full adjustment in Model B (RR = 0.92, 95% CI 0.87–0.98; *p* = 0.013). In contrast, no significant difference in medication burden was observed between the middle tertile (T2) and the lowest tertile in either model.

Taken together, these findings suggest a graded inverse association between diet quality and medication burden, driven primarily by individuals with the highest PHDI adherence, and consistent across alternative model specifications.

### 3.3. Association Between PHDI Score and Prescription of PPIs and NSAIDs

The descriptive association between PHD adherence and decreased consumption of potentially nephrotoxic medications, such as PPIs and NSAIDs, was also investigated through multivariate survey-weighted logistic regression models shown in Table 4.

When PHDI was analyzed as a continuous variable, each 10-point increase in PHDI was associated with significantly lower odds of both PPI and NSAID use. Specifically, higher PHD adherence was associated with 14% lower odds of PPI prescription (OR 0.86, 95% CI 0.79–0.94) and a similarly reduced odds of NSAID use (OR 0.86, 95% CI 0.76–0.97), independent of sociodemographic factors, lifestyle behaviors, comorbidities, kidney function, and albuminuria.

When PHDI was modeled in tertiles, distinct patterns emerged. Compared with participants in the lowest tertile (T1), those in the highest tertile (T3) had substantially lower odds of PPI use (OR 0.66, 95% CI 0.49–0.89). A similar inverse association was observed for NSAID use in T3, although the confidence interval crossed unity (OR 0.67, 95% CI 0.40–1.11). In contrast, individuals in the middle tertile (T2) showed no significant difference in PPI use compared with T1, but exhibited higher odds of NSAID prescription (OR 1.56, 95% CI 1.02–2.40).

Overall, these findings suggest a non-linear relationship between diet quality and the use of potentially nephrotoxic medications, with the most favorable prescribing profile observed among individuals with the highest PHD adherence.

## 4. Discussion

This nationally representative cross-sectional study of older adults with CKD demonstrates that higher adherence to the PHD is independently associated with lower medication burden. Participants in the highest PHDI tertile used approximately 8% fewer medications compared to those in the lowest tertile, even after comprehensive adjustment for sociodemographic factors, lifestyle behaviors, and comorbidities. Each 10-point increase in PHDI score was associated with a 3% reduction in medication count. Importantly, higher PHD adherence was specifically associated with reduced use of potentially nephrotoxic medications, including PPIs and NSAIDs, suggesting that dietary quality may influence medication needs in clinically relevant ways. To our knowledge, this is the first study to examine the relationship between adherence to a sustainable, plant-based dietary pattern and medication burden in older adults with CKD. The findings contribute to growing evidence that dietary interventions may serve as important adjuncts to pharmacological management in CKD, potentially reducing medication complexity and associated risks in vulnerable older populations [13,24].

A central finding of this study was the strong inverse association between PHDI adherence and use of potentially nephrotoxic medications, specifically PPIs and NSAIDs. These two medication classes represented the only significant associations observed in our multivariable analyses, with each 10-point increase in PHDI associated with 14% lower odds of PPI use (OR: 0.86, 95% CI: 0.79–0.94) and 14% lower odds of NSAID use (OR: 0.86, 95% CI: 0.76–0.97). When examined categorically, participants in the highest PHDI tertile demonstrated 34% lower odds of PPI use (OR: 0.66, 95% CI: 0.49–0.89) compared to those in the lowest tertile.

PPIs represent one of the most widely prescribed medication classes globally, with utilization rates particularly high among older adults [35]. In our CKD cohort, overall PPI use was 20.6%, with greater PPI use in the lowest score tertile (21.5%) and the lowest usage in the highest score tertile (16.7%, *p* = 0.007). The association between higher PHD adherence and reduced PPI use likely reflects multiple interconnected pathways related to gastrointestinal health and dietary composition. First, plant-based dietary patterns may directly reduce gastroesophageal reflux symptoms [36]. High consumption of fruits and vegetables provides dietary fiber, which has been associated with lower prevalence of gastroesophageal reflux disease (GERD) in observational studies [36,37]. Additionally, limiting processed foods, red meat, and high-fat items may reduce postprandial gastric acid secretion and lower esophageal sphincter relaxation, key mechanisms underlying GERD pathophysiology. Second, the anti-inflammatory properties of plant-predominant diets may ameliorate underlying gastrointestinal inflammation that contributes to dyspeptic symptoms and reflux [38]. Participants with higher PHDI scores in our cohort exhibited better overall metabolic health, including lower glucose levels and improved HDL-C, suggesting reduced systemic inflammation despite no significant differences in C-reactive protein. Chronic low-grade inflammation has been implicated in functional dyspepsia and may drive PPI prescribing beyond frank GERD [39]. Third, a recent study in a NHANES subcohort has shown that increased PHD adherence is associated with healthier body composition and decreased visceral adiposity [40], the latter being a major risk factor for GERD through increased intra-abdominal pressure and mechanical disruption of the gastroesophageal junction. In any case, the clinical significance of reduced PPI use in CKD populations cannot be overstated. Chronic PPI therapy has been associated with increased risks of acute kidney injury, accelerated CKD progression, electrolyte disturbances (particularly hypomagnesemia), and increased susceptibility to enteric infections, including Clostridioides difficile [35,41].

The inverse association between PHD adherence and NSAID use represents an equally important finding with significant nephrotoxic implications. Overall NSAID use was 8.3% in our cohort, with greater consumption in the lower PHDI score tertile (8.5%) and a lower use in the highest tertile (5.2%, *p* = 0.003). NSAIDs represent a major modifiable risk factor for acute kidney injury, CKD progression, and adverse cardiovascular events, particularly in older adults and those with pre-existing renal impairment [42,43]. Several mechanisms may explain reduced NSAID requirements among individuals with higher PHD adherence. Plant-based dietary patterns possess well-documented anti-inflammatory properties mediated through multiple bioactive compounds, including polyphenols, flavonoids, carotenoids, and omega-3 fatty acids from plant sources. These compounds modulate inflammatory pathways by reducing production of pro-inflammatory cytokines (interleukin-6, tumor necrosis factor-alpha), decreasing oxidative stress, and inhibiting cyclooxygenase and lipoxygenase enzymes, the same enzymatic targets of NSAIDs [44,45]. Consequently, plant-predominant diets may provide natural anti-inflammatory effects that reduce pain and inflammation, thereby decreasing analgesic medication requirements. In this regard, observational studies have demonstrated associations between plant-based dietary patterns and lower prevalence of chronic pain conditions, including osteoarthritis, rheumatoid arthritis, and fibromyalgia [46,47,48]. Additionally, improved metabolic health associated with higher PHD adherence, including better glycemic control and reduced uric acid levels observed in our cohort, may independently reduce inflammatory burden and pain. Hyperglycemia and hyperuricemia both contribute to systemic inflammation and oxidative stress, potentially amplifying pain perception and increasing analgesic requirements. The significant metabolic improvements observed across PHDI tertiles may therefore translate into reduced pain and inflammation, decreasing the clinical indications for NSAID prescribing.

The specificity of associations observed in our study, represented by significant reductions in PPIs and NSAIDs but not in guideline-directed cardiovascular or metabolic therapies, provides important mechanistic insights. This pattern suggests that dietary quality influences medication burden primarily by reducing the need for symptomatic treatments and potentially harmful medications rather than by eliminating evidence-based chronic disease therapies. The convergence of reduced PPI and NSAID use represents a particularly favorable safety profile for older adults with CKD. Both medication classes are frequently prescribed inappropriately or continued unnecessarily beyond initial clinical indications, contributing substantially to the polypharmacy burden. The American Geriatrics Society Beers Criteria identify both chronic PPI use (without documented indication) and NSAIDs as potentially inappropriate medications in older adults, particularly those with CKD [49]. Dietary interventions that reduce reliance on these agents align with deprescribing initiatives and may improve both kidney and overall health outcomes.

Importantly, the observed associations persisted after comprehensive adjustment for comorbidities, kidney function, and other confounders, suggesting that dietary effects on PPI and NSAID use operate through pathways beyond simple correlation with overall health status. Dietary interventions targeting improved diet quality may offer a complementary approach to reducing pharmacological burden while providing additional benefits for overall health and sustainability.

This study has several notable strengths. First, we analyzed data from NHANES, a large, nationally representative survey with rigorous data collection protocols, enhancing generalizability to the broader U.S. population of older adults with CKD. Second, dietary intake was assessed using two 24 h dietary recalls, which provide more detailed information than food frequency questionnaires. Third, medication data were collected through direct observation of medication containers, reducing recall bias. Fourth, we adjusted for numerous potential confounders, including socioeconomic factors, lifestyle behaviors, and comorbidities, strengthening causal inference.

However, important limitations must be acknowledged. The cross-sectional design precludes the establishment of temporal relationships or causality. Reverse causation remains possible; individuals with fewer comorbidities and medications may find it easier to adhere to healthy dietary patterns. Residual confounding cannot be excluded despite comprehensive covariate adjustment. Dietary assessment based on two 24 h recalls may not fully capture long-term dietary patterns and is subject to measurement error and recall bias. Misclassification of medication use and CKD status is possible, although the use of standardized protocols likely minimized such errors. CKD was defined based on single measurements of eGFR and ACR rather than the standard 3-month confirmation period, potentially overestimating CKD prevalence. Finally, our findings may not generalize to non-U.S. populations or to younger adults with CKD.

## 5. Conclusions

In this nationally representative cross-sectional study of U.S. older adults with CKD, higher adherence to the PHD was independently associated with lower medication burden, including reduced use of potentially nephrotoxic medications, such as PPIs and NSAIDs. These findings suggest that plant-based, sustainable dietary patterns may serve as valuable adjuncts to pharmacological management in CKD, potentially reducing medication complexity and associated risks in vulnerable older populations.

## Figures and Tables

**Table 1:** Baseline sociodemographic, clinical, and laboratory characteristics of older adults with CKD, overall and stratified by tertiles of the PHDI.

	Whole (*n* = 3161)	PHDI T1 (*n* = 1054)	PHDI T2 (*n* = 1054)	PHDI T3 (*n* = 1053)	*p* Value
PHDI, mean (SD)	76.6 (12.9)	62.3 (7.1)	77.0 (3.3)	90.5 (6.4)	<0.001
Age, years, mean (SD)	75.0 (5.5)	74.0 (5.6)	75.9 (5.3)	75.2 (5.4)	<0.001
Female gender, *n* (%)	1781 (56.4)	489 (46.3)	602 (57.1)	690 (65.6)	<0.001
White race, *n* (%)	2538 (80.3)	835 (79.1)	828 (78.5)	876 (83.2)	0.024
Poverty income ratio, mean (SD)	2.7 (1.5)	2.5 (1.4)	2.7 (1.5)	3.0 (1.5)	<0.001
Smoking, *n* (%)	1654 (52.3)	648 (61.4)	530 (50.4)	476 (45.2)	<0.001
Alcohol consumption, *n* (%)	1997 (65.0)	701 (68.2)	638 (62.0)	658 (65.0)	0.126
Hypertension, *n* (%)	2321 (73.7)	778 (74.0)	796 (76.0)	746 (71.1)	0.182
Diabetes, *n* (%)	1045 (33.1)	376 (35.6)	351 (33.3)	318 (30.3)	0.187
Heart disease, *n* (%)	789 (25.2)	281 (27.0)	234 (22.4)	274 (26.3)	0.182
Stroke, *n* (%)	380 (12.0)	144 (13.7)	138 (13.2)	98 (9.3)	0.332
Cancer, *n* (%)	944 (29.9)	322 (30.7)	318 (30.1)	304 (28.9)	0.826
COPD, *n* (%)	396 (12.6)	159 (15.2)	122 (11.6)	115 (11.0)	0.115
eGFR, mL/min/1.73 m^2^, mean (SD)	58.3 (18.7)	57.1 (19.2)	58.4 (18.6)	59.3 (18.1)	0.160
ACR, mg/g, median (IQR)	33.0 (9.5–72.5)	34.5 (9.7–74.8)	32.3 (9.0–84.1)	31.5 (10.0–64.2)	0.769
Glucose, mg/dL, mean (SD)	114.1 (41.2)	116.4 (42.7)	115.0 (40.5)	110.9 (40.0)	0.017
HbA1c, %, mean (SD)	6.1 (1.0)	6.2 (1.1)	6.1 (1.1)	6.0 (1.0)	0.058
HDL-C, mg/dL, mean (SD)	54.0 (17.5)	51.0 (17.5)	54.0 (16.4)	57.1 (17.9)	<0.001
LDL-C, mg/dL, mean (SD)	104.2 (38.1)	102.2 (35.6)	106.9 (41.3)	103.2 (36.5)	0.300
CRP, mg/L, mean (SD)	0.6 (1.6)	0.6 (0.9)	0.5 (1.0)	0.7 (2.5)	0.490
Uric acid, mg/dL, mean (SD)	6.2 (1.6)	6.4 (1.6)	6.2 (1.6)	6.1 (1.6)	0.004

Notes: ACR: albumin-to-creatinine ratio; CRP: C-reactive protein; eGFR: estimated glomerular filtration rate; HbA1c: glycated hemoglobin; HDL-C: high-density lipoprotein cholesterol; LDL-C: low-density lipoprotein cholesterol; PHDI: planetary health diet index; poverty income ratio represents the ratio of family income to the federal poverty threshold.

**Table 2:** Medication burden and patterns of medication use in the overall study population and across PHDI tertiles.

	Whole (*n* = 3161)	PHDI T1 (*n* = 1054)	PHDI T2 (*n* = 1054)	PHDI T3 (*n* = 1053)	*p* Value
PHDI, mean (SD)	76.6 (12.9)	62.3 (7.1)	77.0 (3.3)	90.5 (6.4)	<0.001
Number of medications, mean (SD)	5.2 (3.3)	5.4 (3.5)	5.3 (3.3)	4.9 (3.2)	0.025
Polypharmacy, *n* (%)	1703 (53.9)	596 (56.5)	578 (54.8)	529 (50.2)	0.127
ACEi, *n* (%)	1012 (32.0)	340 (32.3)	365 (34.6)	307 (29.1)	0.176
ARBs, *n* (%)	656 (20.8)	200 (18.9)	215 (20.4)	241 (22.9)	0.294
Beta blockers, *n* (%)	1221 (38.6)	414 (39.3)	391 (37.1)	416 (39.5)	0.678
CCBs, *n* (%)	820 (25.9)	293 (27.8)	284 (27.0)	243 (23.1)	0.153
Statins, *n* (%)	1529 (48.4)	519 (49.2)	487 (46.2)	523 (49.7)	0.481
Glucose-lowering medications, *n* (%)	889 (28.1)	322 (30.5)	305 (28.9)	263 (25.0)	0.197
Urate-lowering medications, *n* (%)	190 (6.0)	68 (6.4)	57 (5.4)	65 (6.2)	0.694
Antiplatelet medications, *n* (%)	317 (10.0)	113 (10.7)	114 (10.8)	90 (8.5)	0.268
Phosphate binders, *n* (%)	9 (0.3)	4 (0.4)	1 (0.1)	4 (0.3)	0.319
PPIs, *n* (%)	651 (20.6)	227 (21.5)	248 (23.6)	175 (16.7)	0.007
NSAIDs, *n* (%)	261 (8.3)	90 (8.5)	117 (11.1)	54 (5.2)	0.003

Notes: ACEi: angiotensin converting enzyme inhibitors; ARBs: angiotensin receptor blockers; CCBs: calcium channel blockers; NSAIDs: nonsteroidal anti-inflammatory drugs; PHDI: planetary health diet index; PPIs: proton pump inhibitors.

**Table 3:** Survey-weighted multivariable Poisson regression models showing the association between PHDI, modeled as continuous score (per 10-point increase) and tertiles and number of medications.

	Model A, RR (95%CI) [*p* Value]	Model B, RR (95%CI) [*p* Value]
PHDI-10	0.97 (0.94–0.99) [0.002]	0.97 (0.96–0.99) [0.011]
PHDI T1	-	-
PHDI T2	0.95 (0.88–1.03) [0.23]	0.99 (0.93–1.05) [0.70]
PHDI T3	0.90 (0.83–0.96) [0.003]	0.92 (0.87–0.98) [0.013]

Model A: adjusted for age, gender, race, poverty income ratio; Model B: model A + smoking, alcohol status, hypertension, diabetes, heart disease, stroke, COPD, cancer, eGFR, and ACR; PHDI-10 refers to a 10-point increase in the Planetary Health Diet Index score.

**Table 4:** Survey-weighted multivariable logistic regression models of PPI and NSAID use according to PHDI, modeled as a continuous score (per 10-point increase) and tertiles.

	PPI Use	NSAID Use
	Model B_1_, OR (95%CI)	Model B_2_, OR (95%CI)
PHDI-10	0.86 (0.79–0.94)	0.86 (0.76–0.97)
PHDI T1	-	-
PHDI T2	1.14 (0.87–1.50)	1.56 (1.02–2.40)
PHDI T3	0.66 (0.49–0.89)	0.67 (0.40–1.11)

Model B_1_: adjusted for age, gender, race, poverty income ratio, smoking, alcohol status, hypertension, diabetes, heart disease, stroke, COPD, cancer, eGFR, ACR, and NSAIDs; model B_2_: adjusted for age, gender, race, poverty income ratio, smoking, alcohol status, hypertension, diabetes, heart disease, stroke, COPD, cancer, eGFR, ACR, and PPIs; PHDI-10 refers to a 10-point increase in the Planetary Health Diet Index score.

## Data Availability

Publicly available datasets were analyzed in this study. These data can be found at the National Health and Nutrition Examination Survey website: https://wwwn.cdc.gov/nchs/nhanes/ (accessed on 15 December 2025).

## Supplementary Materials

The following supporting information can be downloaded at: https://www.mdpi.com/article/10.3390/geriatrics11010017/s1, Table S1: PHDI components and criteria for definition of scoring system.

## Abbreviations

The following abbreviations are used in this manuscript:

ACEi	Angiotensin-converting enzyme inhibitors
ACR	Albumin-to-creatinine ratio
ARBs	Angiotensin receptor blockers
CCBs	Calcium channel blockers
CKD	Chronic kidney disease
COPD	Chronic obstructive pulmonary disease
CRP	C-reactive protein
eGFR	Estimated glomerular filtration rate
HbA1c	Glycated hemoglobin
HDL-C	High-density lipoprotein cholesterol
LDL-C	Low-density lipoprotein cholesterol
NHANES	National Health and Nutrition Examination Survey
NSAIDs	Nonsteroidal anti-inflammatory drugs
PHD	Planetary Health Diet
PHDI	Planetary Health Diet Index
PPIs	Proton pump inhibitors
