# Supplementary material for: Planetary Health Diet Adherence and Medication Use in Older Adults with Chronic Kidney Disease: A Cross-Sectional Study
Source: Geriatrics (Basel). 2026 Feb 5;11(1):17. doi: 10.3390/geriatrics11010017 (PMC12921894; doi:10.3390/geriatrics11010017)
Supplement: Supplementary file 1 [file geriatrics-11-00017-s001.zip › geriatrics-4107683-supplementary.pdf]

Supplementary Table S1. PHDI components and criteria for definition of scoring system

	EAT-Lancet reference diet		Scoring criteria		
	**Grams/day**	**Kcal/day**	**Min score (0) in g/d**	**Max score (10) in g/d**	**Weighted score**
**Adequacy**					
Whole grains	232 (0–60% of TEI)	811	0	≥ 75 g/d in M; ≥ 90 g/d in F	1
Non-starchy vegetables	300 (200–600)	78	0	≥ 300	1
Whole fruits	200 (100–300)	126	0	≥ 200	1
Soybean and soy foods	25 (0–50)	112	0	≥ 50	0.5
Non soy legumes (e.g dry beans, peas, lentils)	50 (0–100)	172	0	≥ 100	0.5
Nuts (e.g. peanuts and tree nuts)	50 (0–75)	291	0	≥ 50	1
Fish and shellfish	28 (0–100)	40	0	≥ 28	1
Added unsaturated oils (e.g. olive soybean, rapeseed, peanut oil, sunflower oil)	40 (20–80)	354 (14.16 % of TEI)	≤ 3.5 % of TEI	≥ 21 % of TEI	1
**Moderation**					
Tubers and starchy vegetables	50 (0–100)	39	≥ 200	≤ 50	1
Dairy	250 (0–500)	153	≥ 1000	≤ 250	1
Eggs	13 (0–25)	19	≥ 120	≤ 13	1
Red and processed meat (e.g. beef, pork, lamb)	14 (0–28)	30	100	≤ 14	1
Poultry (e.g. chicken, duck, goose, ostrich)	29 (0–58)	62	≥ 100	≤ 29	1
Added saturated fats (e.g. palm oil, coconut oil, dairy fat-butter, margarine, lard, tallow)	11.8 (0–11.8)	96 (3.8 % of TEI)	≥ 10 % of TEI	0 % of TEI	1
Added sugars and fruit juices	31 (0–31)	120 (4.8 % of TEI)	≥ 25 % of TEI	≤ 5 % of TEI	1

Notes: TEI: total energy intake.
